# Nutritional biomarkers and heart failure requiring hospitalization in patients with type 2 diabetes: the SURDIAGENE cohort

**DOI:** 10.1186/s12933-022-01505-9

**Published:** 2022-06-09

**Authors:** Matthieu Wargny, Mikaël Croyal, Stéphanie Ragot, Elise Gand, David Jacobi, Jean-Noël Trochu, Xavier Prieur, Cédric Le May, Thomas Goronflot, Bertrand Cariou, Pierre-Jean Saulnier, Samy Hadjadj, for the SURDIAGENE study group, Richard Marechaud, Vincent Javaugue, Charlotte Hulin-Delmotte, Pierre Llatty, Gregory Ducrocq, Ronan Roussel, Vincent Rigalleau, Yann Pucheu, David Montaigne, Jean-Michel Halimi, Philippe Gatault, Philippe Sosner, Barnabas Gellen

**Affiliations:** 1 Nantes Université, CHU Nantes, CNRS, INSERM, l’institut du thorax, 44000 Nantes, France; 2 CHU de Nantes, INSERM CIC 1413, Pôle Hospitalo-Universitaire 11: Santé Publique, Clinique des données, Nantes, France; 3 Université de Poitiers, INSERM CHU de Poitiers, Centre d’Investigation Clinique, CIC 1402, Poitiers, France; 4 Université de Nantes, CHU Nantes, Inserm, CNRS, SFR Santé, Inserm UMS 016, CNRS UMS 3556, 44000 Nantes, France; 5 CRNH-Ouest Mass Spectrometry Core Facility, 44000 Nantes, France; 6 Nantes Université, CNRS, INSERM, l’institut du thorax, 44000 Nantes, France

**Keywords:** TMAO, Nutritional biomarkers, Diabetes mellitus, Heart failure, Cohort study, Homocysteine

## Abstract

**Background:**

Heart failure (HF) is a growing complication and one of the leading causes of mortality in people living with type 2 diabetes (T2D). Among the possible causes, the excess of red meat and the insufficiency of vegetables consumption are suspected. Such an alimentation is associated with nutritional biomarkers, including trimethylamine *N*-oxide (TMAO) and its precursors. Here, we aimed to study these biomarkers as potential prognostic factors for HF in patients with T2D.

**Methods:**

We used the SURDIAGENE (SURvival DIAbetes and GENEtics) study, a large, prospective, monocentric cohort study including 1468 patients with T2D between 2001 and 2012. TMAO and its precursors (trimethylamine [TMA], betaine, choline, and carnitine) as well as thio-amino-acids (cysteine, homocysteine and methionine) were measured by liquid chromatography-tandem mass spectrometry. The main outcome was HF requiring Hospitalization (HFrH) defined as the first occurrence of acute HF leading to hospitalization and/or death, established by an adjudication committee, based on hospital records until 31st December 2015. The secondary outcomes were the composite event HFrH and/or cardiovascular death and all-cause death. The association between the biomarkers and the outcomes was studied using cause-specific hazard-models, adjusted for age, sex, history of coronary artery disease, NT-proBNP, CKD-EPI-derived eGFR and the urine albumin/creatinine ratio. Hazard-ratios (HR) are expressed for one standard deviation.

**Results:**

The data of interest were available for 1349/1468 of SURDIAGENE participants (91.9%), including 569 (42.2%) women, with a mean age of 64.3 ± 10.7 years and a median follow-up of 7.3 years [25th–75th percentile, 4.7–10.8]. HFrH was reported in 209 patients (15.5%), HFrH and/or cardiovascular death in 341 (25.3%) and all-cause death in 447 (33.1%). In unadjusted hazard-models, carnitine (HR = 1.20, 95% CI [1.05; 1.37]), betaine (HR = 1.34, [1.20; 1.50]), choline (HR = 1.35, [1.20; 1.52]), TMAO (HR = 1.32, [1.16; 1.50]), cysteine (HR = 1.38, [1.21; 1.58]) and homocysteine (HR = 1.28, [1.17; 1.39]) were associated with HFrH, but not TMA and methionine. In the fully adjusted models, none of these associations was significant, neither for HFrH nor for HFrH and/or CV death, when homocysteine only was positively associated with all-cause death (HR = 1.16, [1.06; 1.27]).

**Conclusions:**

TMAO and its precursors do not appear to be substantial prognosis factors for HFrH, beyond usual cardiac- and kidney-related risk factors, whereas homocysteine is an independent risk factor for all-cause death in patients with T2D.

**Supplementary Information:**

The online version contains supplementary material available at 10.1186/s12933-022-01505-9.

## Introduction

Diabetes is a globally-increasing condition and is expected to affect more than 10% of humans worldwide in 2030 [1]. In addition to microvascular complications, cardiovascular disease is one of the leading causes of morbidity and mortality in patients with diabetes [2, 3]. Subjects living with diabetes present an excess risk of coronary artery disease (CAD) and diabetic cardiomyopathy, which are the two main causes of heart failure (HF). Recently, a large meta-analysis considering over 12 million individuals evidenced a two-fold greater risk of HF in individuals with type 2 diabetes (T2D) compared to those without, both in men and women [4].

Among the multiple factors associated with such complex diseases, nutrition appears to be essential since it is a possible target for preventive and/or therapeutic actions, as a modifiable risk factor for both T2D and HF. In that respect, glycemic index and load were recently shown to be related to cardiovascular disease in a large-scale global epidemiological approach. However, when scrutinizing the effect of different outcomes, it turned out that glycemic index and load were associated with atherosclerosis-related cardiovascular events but not with HF [5].

So far, the impact of nutrition in HF is often under-estimated. In the guidelines of the ESC (European Society of Cardiology), nutrition in HF mainly relates to malnutrition and obesity as contributor of HF. Dietary recommendations are mainly focused on salt intake, healthy eating, maintenance of body weight and refraining from excessive alcohol intake in case of toxic cardiomyopathy [6]. We previously evidenced the association between markers of red meat consumption (trimethylamine *N*-oxide [TMAO] and related metabolites) and the occurrence of major adverse cardiovascular events (MACE), but also mortality in patients with T2D [7]. However, we did not evaluate the impact of nutritional biomarkers on HF, a critical outcome in persons living with diabetes.

A 2019 initiative, the EAT-Lancet commission on healthy diet, recommended to consider 14 key items for universal healthy diet [8]. Being able to identify nutritional biomarkers associated with HF is an important issue especially in T2D. This could in particular enable the early identification of patients susceptible to develop HF and for whom specific management including nutritional counselling could be considered in a preventive manner. Such an approach could pave the way for personalized nutrition of patients with T2D.

Thus, we aimed to assess how baseline nutritional biomarkers related to red meat intake (TMAO and related compounds, i.e. trimethylamine [TMA], betaine, choline and carnitine) and to vegetable intakes (thio-amino-acids: cysteine, homocysteine and methionine) were associated with the incidence of HF requiring Hospitalization (HFrH) in patients with T2D, regardless of their history of HF.

## Methods

### SURDIAGENE cohort and study population

The design of the SURvival DIAbetes and GENEtics (SURDIAGENE) cohort has already been described elsewhere [9]. Briefly, SURDIAGENE is a large, prospective, monocentric cohort study with the consecutive inclusion of 1468 T2D patients taken care at the Diabetes Department at Poitiers University Hospital, France, between 2001 and 2012. The study was primarily designed to identify the genetic determinants of micro- and macrovascular diabetic complications. At baseline, clinical and biological data were collected and blood/urine samples were drawn. Clinical events corresponding to endpoints of interest were collected during follow-up, based on consultations with general practitioner and hospital records.

Renal function was assessed using estimated glomerular filtration rate (eGFR) calculated with the CKD-EPI 2009-formula [10]. For the present analysis, we excluded patients with: baseline eGFR < 30 mL/min. 1.73 m^2^; renal replacement therapy (need for dialysis or history of renal transplant); missing data for ≥ 1 of the following: NT-proBNP, urine albumin/creatinine ratio (uACR), nutritional biomarkers of red meat intake (methylamines: carnitine, betaine, choline, TMAO, TMA) and of vegetable intake (amino-acids: cysteine, homocysteine and methionine).

### Definition of clinical history

History of CAD was defined as history of angina pectoris and/or coronary revascularization and/or myocardial infarction. Cerebrovascular disease (CVD) was defined as history of stroke and/or transient ischemic attack. Lower limb artery disease was defined as lower limb revascularization and/or amputation.

### Biology assays

The methylamines (TMAO and its precursors, TMA, betaine, choline and carnitine) as well as the thio-amino-acids (cysteine, homocysteine and methionine) were analysed in baseline fasting plasma samples by liquid chromatography-tandem mass spectrometry as detailed in Additional file 1. Samples were stored at − 80 °C until final use with only 2 freeze/thaw cycle. The intra- and inter-assay imprecisions of the analytical method were assessed throughout experiments and were below 10.2% for all compounds. All compounds were found stable in a set sample of 10 patients with diabetes provided by CHU Nantes (“*maladies métaboliques*” collection) after 3 freeze/thaw cycles with mean recovery ranging from 93.7% to 111.1%. At completion of the study, a representative set of samples (~ 10% of the cohort) was arbitrarily re-analysed 6 months after the initial determination. The new plasma concentrations did not vary by more than 7.2% (from − 6.8% to 7.2%; median: 3.2% [− 4.3%; 5.5%]) in comparison with the first analysis.

NT-proBNP was measured in baseline plasma-EDTA samples by an electrochemiluminescence automated assay (Roche Diagnostics, Mannheim, Germany).

### Outcomes

The primary outcome of the study was the first occurrence of HFrH during follow-up. HFrH was defined as the first occurrence of one of the following events, whichever came first: acute HF requiring hospitalization or leading to death, validated by an adjudication committee including both diabetologists and cardiologists, after careful evaluation of hospital and discharge records. We proposed the study of two secondary outcomes: (i) all-cause death, established after linking French national death registry in SURDIAGENE participants; and (ii) HFrH and/or cardiovascular (CV) death, the latter validated by an adjudication committee. Follow-up data were collected until 31st December, 2015.

### Statistical analyses

For baseline analysis, patients’ characteristics were presented as numbers (%) for categorical parameters, and mean ± SD or median (25th–75th percentile) for quantitative parameters. They were compared according to the final follow-up event for HFrH and all-cause death. Independence between categorical parameters was tested using Fisher’s exact test. For testing difference for quantitative parameters between two groups, we proposed Student’s t-test or Mann–Whitney U-test according to variable distribution, as deemed appropriate.

For longitudinal analysis, we proposed 5 models for each nutritional biomarker of interest, with different adjustment levels. The nutritional biomarkers were tested one-by-one, separately. Model 1 (M_1_): biomarker only; Model 2 (M_2_): M_1_ adjusted for age and sex; Model 3A (M_3A_): M_2_ adjusted for cardiac covariates (history of CAD and NT-proBNP); Model 3B (M_3B_): M_2_ adjusted for renal covariates (eGFR and uACR); Model 4 (M_4_, full model): M_2_ adjusted for both cardiac and renal covariates. We considered all-cause death as a competing risk in the analysis of HFrH and followed the recommendation summarized by Austin et al. [11]. So, we calculated HR for (i) cause-specific hazards using Cox regression models, and (ii) relative incidences, using subdistribution hazard models [12]. For quantitative parameters, log-transformation was applied when appropriate and HR were calculated for an increase of 1 SD. Additionally, plots of the cumulative incidence functions (CIF, here with a quartile-based approach) are proposed for HFrH and all-cause death.

A global p-value < 0.05 was considered as statistically significant. Considering 8 parameters studied in 3 main analyses (survival for HFrH, HFrH and/or CV death, and all-cause death), and disregarding the different models used (M1 to M4, cause-specific and subdistribution hazards models) considered as heavily correlated, we proposed a conservative threshold = 0.0021 (≈0.05/24) for individual p-value, following a Bonferroni approach.

We challenged the linearity assumption using fractional polynomials of degree 2 (FP2) [13] to test for other potential shapes of the HR function linking each biomarker and HFrH. However, even with an alpha value for FP2 as high as 0.50, no transformation was proposed, supporting therefore the linearity.

As an exploratory analysis, we also proposed subgroups analyses of the population according to baseline status for CAD, obesity and NT-proBNP level (below or above 125 pg/mL), using cause-specific hazard models adjusted for age and sex for the study of HFrH.

All results are presented using available data, without imputation. All statistical analyses were performed using R version 4.0.0., particularly with “*cpmrsk”* package [14, 15].

## Results

**Fig. 1:**
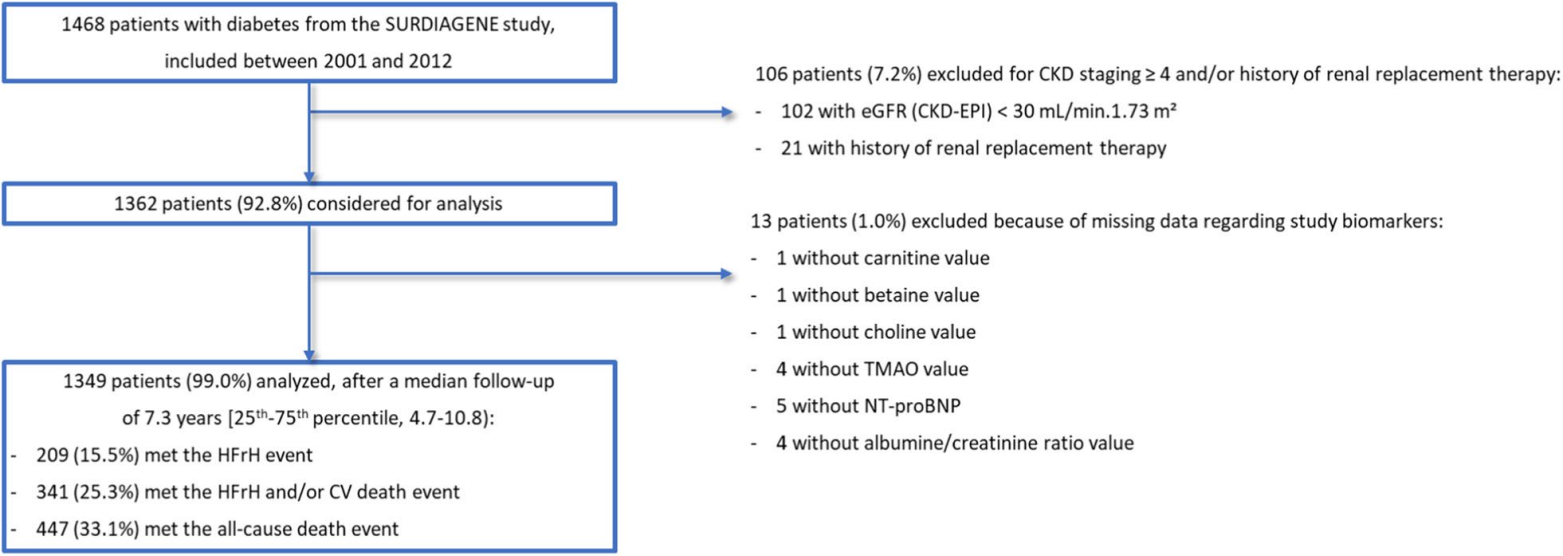
Study flow-chart. CKD: Chronic Kidney Disease; CV: Cardiovascular; HFrH: Heart Failure requiring Hospitalization, defined as the first occurrence of acute HF leading to hospitalization and/or death; NT-proBNP: N-terminal prohormone of brain natriuretic peptide; TMAO: trimethylamine *N*-oxide

The SURDIAGENE study included 1468 patients with T2D, of which 106 patients (7.2%) were secondarily excluded because of eGFR < 30 mL/min. 1.73 m^2^ and/or renal replacement therapy at baseline. In order to ensure data consistency and nested multivariable regression models, 13 patients (1.0%) were also removed from the present analysis because of missing data for methylamines and/or NT-proBNP and/or uACR. Finally, 1349 patients (91.9%) were included in the present analysis, with a median follow-up of 7.3 years [25th–75th percentile, 4.7–10.8]. HFrH occured in 209 patients (15.5%), HFrH and/or CV death in 341 patients (25.3%) and all-cause death in 447 patients (33.1%). Flow-chart details can be found in Fig. 1.

A comparison between baseline characteristics according to the occurrence of the outcomes is proposed Table 1. Patients who met the HFrH event were older (70.3 ± 9.4 vs 63.2 ± 10.5 years, p < 0.0001) and had a lower body mass index (BMI, 30.4 ± 6.1 vs 31.6 ± 6.3 kg/m^2^, p = 0.012) than patients without HFrH. They presented more characteristics of microangiopathy, with a greater proportion of macroalbuminuria (32.0 vs 16.5%, p < 0.001), a lower eGFR (66.1 ± 20.1 vs 78.7 ± 20.5 mL/min. 1.73 m^2^, p < 0.0001) and a greater proportion of severe non-proliferative or proliferative retinopathy (21.8 vs. 11.1%, p < 0.0001). They also presented a greater proportion of macroangiopathy-related history, such as CAD (50.2 vs 27.2%, p < 0.0001), CVD (17.7 vs 11.8%, p = 0.024) and lower limb artery disease (17.2 vs 6.7%, p < 0.0001).

**Table 1:** Baseline characteristics of the cohort classified by status for HFrH and all-cause death during follow-up

Baseline characteristics	All (n = 1349)	Event: HFrH			Event: All-cause death		
		No (n = 1140)	Yes (n = 209)	P-value	No (n = 902)	Yes (n = 447)	P-value
Sex (female)	569/1349 (42.2%)	480/1140 (42.1%)	89/209 (42.6%)	0.94	414/902 (45.9%)	155/447 (34.7%)	< 0.0001
Age (y)	64.3 ± 10.7	63.2 ± 10.5	70.3 ± 9.4	< 0.0001	61.5 ± 10.4	70 ± 8.8	< 0.0001
Weight (kg)	86.4 ± 18.5	87.2 ± 18.5	81.9 ± 17.8	< 0.0001	87.2 ± 18.5	84.8 ± 18.5	0.025
BMI (kg/m.^2^)	31.4 ± 6.3	31.6 ± 6.3	30.4 ± 6.1	0.012	31.7 ± 6.3	30.8 ± 6.2	0.012
Diabetes duration (y)	12 [6; 20]	11 [5; 19]	18 [12; 27]	< 0.0001	11 [5; 17]	16 [10; 25]	< 0.0001
HbA_1c_ (%)	7.8 ± 1.6	7.8 ± 1.6	7.9 ± 1.4	0.61	7.8 ± 1.6	7.9 ± 1.5	0.15
Smoker				0.0090			0.37
Never	689/1331 (51.8%)	584/1125 (51.9%)	105/206 (51.0%)		471/890 (52.9%)	218/441 (49.4%)	
Former	493/1331 (37.0%)	404/1125 (35.9%)	89/206 (43.2%)		318/890 (35.7%)	175/441 (39.7%)	
Active	149/1331 (11.2%)	137/1125 (12.2%)	12/206 (5.8%)		101/890 (11.3%)	48/441 (10.9%)	
Heart rate (bpm)	70.9 ± 13.6	70.9 ± 13.5	71.0 ± 14.0	0.90	70.5 ± 13.1	71.6 ± 14.4	0.19
Systolic BP (mmHg)	132 ± 17.4	131.4 ± 16.8	135.1 ± 19.8	0.013	130.1 ± 16.5	135.7 ± 18.5	< 0.0001
Diastolic BP (mmHg)	72.5 ± 11.1	72.7 ± 10.9	71.0 ± 12.2	0.059	72.6 ± 11	72.2 ± 11.4	0.47
Albuminuria stage				< 0.0001			< 0.0001
Normal to mildly increased	547/1204 (45.4%)	482/1010 (47.7%)	65/194 (33.5%)		423/797 (53.1%)	124/407 (30.5%)	
Moderately increased	428/1204 (35.5%)	361/1010 (35.7%)	67/194 (34.5%)		273/797 (34.3%)	155/407 (38.1%)	
Severely increased	229/1204 (19.0%)	167/1010 (16.5%)	62/194 (32.0%)		101/797 (12.7%)	128/407 (31.4%)	
uACR (mg/mmol)	3 [1; 10]	2 [1; 9]	7 [2; 31]	< 0.0001	2 [1; 7]	7 [2; 30]	< 0.0001
eGFR (CKD-EPI, mL/min/1.73 m.^2^)	76.7 ± 21.0	78.7 ± 20.5	66.1 ± 20.4	< 0.0001	80.8 ± 19.7	68.5 ± 21.1	< 0.0001
Coronary artery disease	364/1349 (27.0%)	259/1140 (22.7%)	105/209 (50.2%)	< 0.0001	187/902 (20.7%)	177/447 (39.6%)	< 0.0001
Cerebrovascular disease	172/1349 (12.8%)	135/1140 (11.8%)	37/209 (17.7%)	0.024	88/902 (9.8%)	84/447 (18.8%)	< 0.0001
Carotid revascularisation	30/1349 (2.2%)	22/1140 (1.9%)	8/209 (3.8%)	0.12	16/902 (1.8%)	14/447 (3.1%)	0.12
Lower limb artery disease	112/1349 (8.3%)	76/1140 (6.7%)	36/209 (17.2%)	< 0.0001	44/902 (4.9%)	68/447 (15.2%)	< 0.0001
Total cholesterol (mmol/L)	4.78 ± 1.14	4.78 ± 1.15	4.75 ± 1.11	0.70	4.75 ± 1.09	4.83 ± 1.24	0.23
LDL-c (mmol /L)	2.73 ± 0.95	2.75 ± 0.96	2.65 ± 0.88	0.14	2.72 ± 0.93	2.77 ± 0.99	0.39
HDL-c (mmol /L)	1.21 ± 0.41	1.20 ± 0.40	1.26 ± 0.46	0.065	1.21 ± 0.39	1.20 ± 0.46	0.68
Triglycerides (mmol/L)	1.89 ± 1.43	1.88 ± 1.44	1.91 ± 1.37	0.81	1.85 ± 1.22	1.96 ± 1.77	0.28
NT-proBNP (pg/mL)	102 [47; 261]	82 [41; 202]	339 [161; 828]	< 0.0001	70 [36; 165]	234 [97; 578]	< 0.0001

Data are expressed using number (%) for categorical data, and mean ± SD or median [25th–75th percentile] for quantitative data, as appropriate. P-values are calculated using Fisher’s exact test for categorical data, and Student T-test or Mann–Whitney U-test for quantitative data

Coronary artery disease was defined as any of the following: angina, coronary revascularization, myocardial infarction. Cerebrovascular disease was defined as any of the following: stroke, transient ischaemic attack. Lower limb artery disease was defined as lower limb revascularization and/or amputation

BMI: Body Mass Index; BP: blood pressure; CKD: Chronic Kidney Disease. HDL-c: high-density-lipoprotein-cholesterol; HFrH: Heart Failure requiring Hospitalization, defined as the first occurrence of acute HF leading to hospitalization and/or death; LDL-c: low-density-lipoprotein-cholesterol; NT-proBNP: N-terminal prohormone of brain natriuretic peptide; uACR: urine albumin/creatinine ratio

The relationship between the baseline concentrations of nutritional biomarkers of dietary components is available in Additional file 2: Fig. S1. As expected, betaine and choline concentrations were positively correlated, as well as homocysteine and cysteine concentrations.

Regarding methylamines-related biomarkers, compared to participants who did not develop HFrH, those who developed HFrH had higher concentrations of carnitine (45.8 ± 14.8 µmol/L vs. 43.7 ± 12.0, p = 0.044), betaine (36.5 ± 16.1 µmol/L vs. 33.0 ± 13.6, p = 0.0030), choline (1.57 ± 0.43 µmol/L vs. 1.46 ± 0.37, p < 0.0001) and TMAO (8.8 µmol/L [5.3; 17.0] vs. 6.6 [4.0; 12.3], p < 0.0001), but not for TMA (0.75 ± 0.27 vs. 0.77 ± 0.27) (Table 2).

**Table 2:** Baseline values for the nutritional biomarkers of interest, classified by status for follow-up events

Baseline characteristics	All (n = 1349)	Event: HFrH			Event: All-cause death		
		No (n = 1140)	Yes (n = 209)	P-value	No (n = 902)	Yes (n = 447)	P-value
Methylamines							
Carnitine (µmol/L)	44.0 ± 12.4	43.7 ± 12.0	45.8 ± 14.8	0.044	43.9 ± 12.0	44.2 ± 13.3	0.66
Betaine (µmol/L)	33.5 ± 14.1	33.0 ± 13.6	36.5 ± 16.1	0.0030	32.5 ± 13.0	35.5 ± 15.8	0.001
Choline (µmol/L)	1.48 ± 0.38	1.46 ± 0.37	1.57 ± 0.43	< 0.0001	1.44 ± 0.34	1.54 ± 0.44	< 0.0001
TMAO (µmol/L)	6.8 [4.2; 12.8]	6.6 [4.0; 12.3]	8.8 [5.3; 17.0]	< 0.0001	6.5 [4.0; 11.7]	7.9 [4.8; 15.3]	< 0.0001
TMA (µmol/L)	0.76 ± 0.27	0.77 ± 0.27	0.75 ± 0.27	0.53	0.76 ± 0.26	0.76 ± 0.28	0.93
Thio-amino-acids							
Cysteine (µmol/L)	23 [13; 39]	23 [13; 39]	23 [14; 40]	0.38	24 [13; 40]	21 [14; 37]	0.57
Homocysteine (µmol/L)	8.9 [4.7; 14.8]	8.6 [4.7; 14.1]	10.9 [5.7; 18.0]	< 0.0001	8.2 [4.5; 13.5]	10.9 [5.7; 17.5]	< 0.0001
Methionine (µmol/L)	26.1 ± 7.1	26.1 ± 6.8	26.1 ± 8.2	0.93	26.3 ± 6.8	25.7 ± 7.5	0.20

Data are expressed using mean ± SD or median [25th–75th percentile] for quantitative data, as appropriate. P-values are calculated using Fisher’s exact test for categorical data, and Student T-test or Mann–Whitney U-test for quantitative data

HFrH: Heart Failure requiring Hospitalization, defined as the first occurrence of acute HF leading to hospitalization and/or death; TMA: trimethylamine; TMAO: trimethylamine *N*-oxide

Regarding nutritional biomarkers of vegetable intake, among the 3 considered thio-amino-acids, homocysteine was higher in patients ultimately yielding HFrH (10.9 µmol/L [5.7; 18.0] vs. 8.6 [4.7; 14.1], p < 0.0001), but no statistical difference was found for cysteine and methionine concentration.

**Fig. 2:**
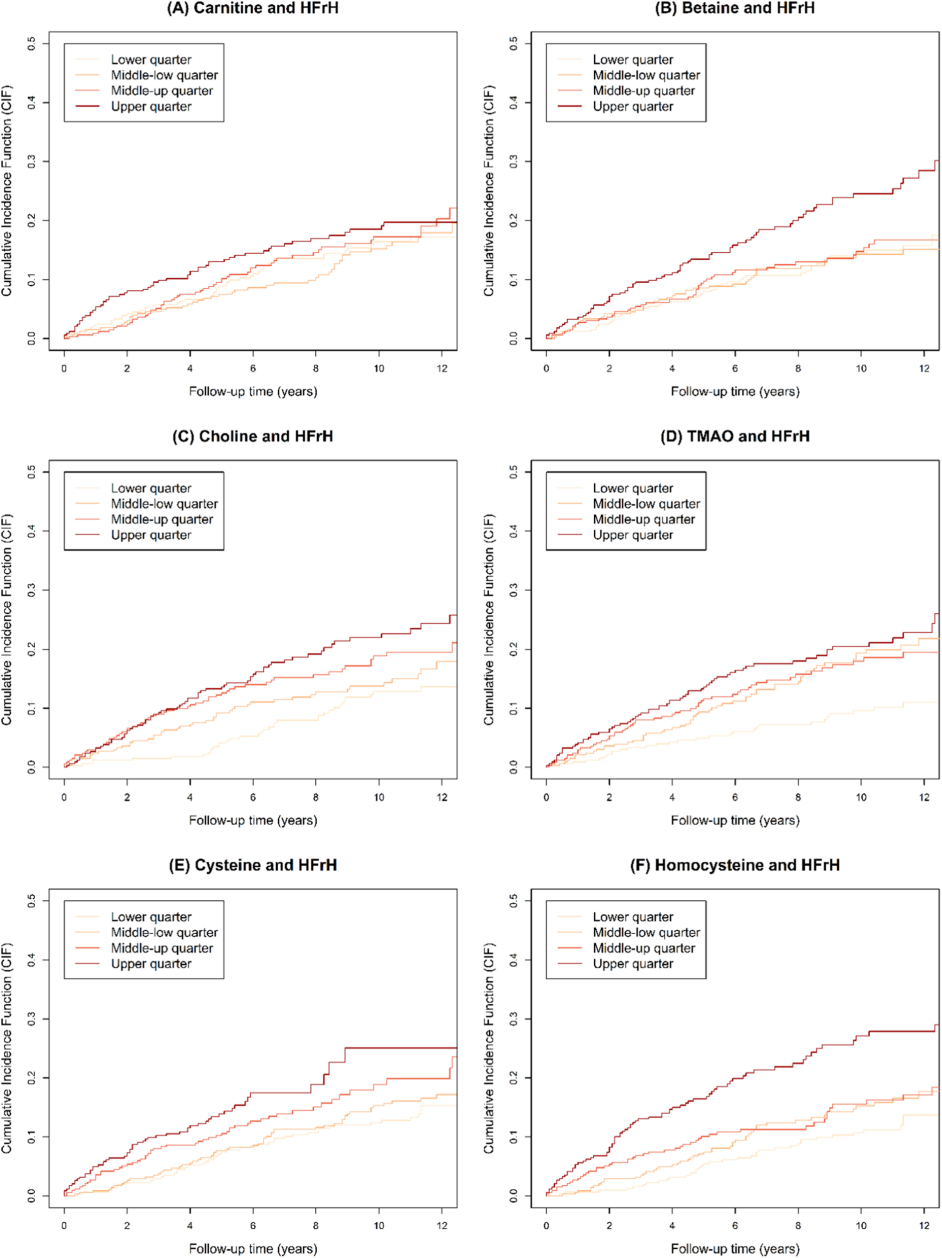
Cumulative Incidence Function for HFrH. Quartile values for the different parameters of interest: carnitine (median = 42.6, [25th–75th] percentile = [35.4–50.7]); betaine (31.5, [24.6–38.9]); choline (1.43, [1.22–1.68]); TMAO (6.8, [4.2–12.8]); cysteine (23, [13–39]); homocysteine (8.9, [4.7–14.8]). HFrH: Heart Failure requiring Hospitalization, defined as the first occurrence of acute HF leading to hospitalization and/or death; TMAO: trimethylamine *N*-oxide

The results of the cause-specific hazard models for HFrH, HFrH and/or CV death and all-cause death are presented in Table 3, and the subdistribution hazard models are presented in Additional file 2 Table S1. In unadjusted hazard-models, carnitine (HR = 1.20, 95% CI [1.05; 1.37], p = 0.0065), betaine (HR = 1.34, [1.20; 1.50], p < 0.0001), choline (HR = 1.35, [1.20; 1.52], p < 0.0001), TMAO (HR = 1.32, [1.16; 1.50], p < 0.0001), cysteine (HR = 1.38, [1.21; 1.58], p < 0.0001) and homocysteine (HR = 1.28, [1.17; 1.39], p < 0.0001) were associated with HFrH, but not TMA and methionine. These associations remained significant after adjustment for age and sex. After further adjustment for eGFR and uACR, only betaine remained statistically associated with HFrH (HR = 1.33, [1.17; 1.50], p < 0.0001). In the fully adjusted models, none of these associations remained significant. The curves for cumulative incidence functions for HFrH are plotted in Fig. 2, and Additional file 2: Fig. S2 for all-cause death. For betaine and homocysteine, the increased risk of HFrH seemed associated with the upper quarter of concentrations.

**Table 3:** Survival analysis for HFrH, the composite HFrH and/or CV death event and all-cause death

	Unadjusted model		Fully adjusted model	
	HR (95_%CI_)	*P*-value	HR (95_%CI_)	*P*-value
Cause-specific HM for HFrH				
Carnitine	1.20 [1.05; 1.37]	0.0065	1.13 [0.99; 1.29]	0.061
Betaine	1.34 [1.20; 1.50]	< 0.0001	1.11 [0.97; 1.27]	0.13
Choline	1.35 [1.20; 1.52]	< 0.0001	0.94 [0.82; 1.08]	0.39
TMAO*	1.32 [1.16; 1.50]	< 0.0001	1.09 [0.94; 1.26]	0.24
TMA	1.01 [0.89; 1.15]	0.86	0.98 [0.86; 1.13]	0.78
Cysteine	1.38 [1.21; 1.58]	< 0.0001	1.10 [0.95; 1.28]	0.20
Homocysteine	1.28 [1.17; 1.39]	< 0.0001	1.05 [0.91; 1.21]	0.49
Methionine	1.02 [0.89; 1.18]	0.73	1.06 [0.93; 1.22]	0.38
Cause-specific HM for HFrH and/or CV death				
Carnitine	1.12 [1.01; 1.25]	0.037	1.06 [0.95; 1.17]	0.32
Betaine	1.27 [1.16; 1.40]	< 0.0001	1.04 [0.93; 1.17]	0.46
Choline	1.28 [1.17; 1.42]	< 0.0001	0.91 [0.82; 1.02]	0.093
TMAO*	1.31 [1.19; 1.45]	< 0.0001	1.10 [0.98; 1.23]	0.11
TMA	1.02 [0.93; 1.13]	0.65	0.99 [0.89; 1.10]	0.81
Cysteine	1.31 [1.17; 1.46]	< 0.0001	1.04 [0.92; 1.18]	0.49
Homocysteine	1.28 [1.20; 1.37]	< 0.0001	1.08 [0.97; 1.21]	0.16
Methionine	0.96 [0.85; 1.08]	0.46	1.00 [0.89; 1.11]	0.93
Cause-specific HM for all-cause death				
Carnitine	1.05 [0.95; 1.15]	0.32	1.01 [0.92; 1.11]	0.84
Betaine	1.28 [1.18; 1.39]	< 0.0001	1.07 [0.97; 1.18]	0.20
Choline	1.26 [1.16; 1.38]	< 0.0001	0.94 [0.85; 1.04]	0.23
TMAO*	1.20 [1.10; 1.31]	< 0.0001	1.03 [0.94; 1.14]	0.52
TMA	1.05 [0.97; 1.14]	0.26	1.01 [0.93; 1.10]	0.83
Cysteine	1.34 [1.22; 1.48]	< 0.0001	1.08 [0.97; 1.20]	0.15
Homocysteine	1.30 [1.23; 1.38]	< 0.0001	1.16 [1.06; 1.27]	0.0011
Methionine	0.96 [0.87; 1.06]	0.37	0.98 [0.89; 1.07]	0.66

^*^TMAO was natural-log transformed before standardization. Cause-specific hazard models were fitted using two adjustment models, unadjusted and fully adjusted. Covariates added in the fully adjusted models were age, sex, history of CAD, log transformed NT-proBPNP, eGFR and log transformed uACR. The nutritional biomarkers were tested separately from each other in the different adjustment models. All HR are given per 1 SD of the given parameter

Abbreviations: CV: cardiovascular; eGFR: estimated glomerular filtration rate calculated with the CKD-EPI 2009-formula; HFrH: Heart Failure requiring Hospitalization, defined as the first occurrence of acute HF leading to hospitalization and/or death; HM: Hazard model; HR: Hazard-ratio; NT-proBNP: N-terminal prohormone of brain natriuretic peptide; TMA: trimethylamine; TMAO: trimethylamine N-oxide; uACR: urine albumin/creatinine ratio

When studying the risk of all-cause death, betaine (HR = 1.28, [1.18; 1.39], p < 0.0001), choline (HR = 1.26, [1.16; 1.38], p < 0.0001), TMAO (HR = 1.20, [1.10; 1.31], p < 0.0001), cysteine (HR = 1.34, [1.22; 1.48], p < 0.0001) and homocysteine (HR = 1.30, [1.23; 1.38], p < 0.0001) were associated with all-cause death in univariate model, but not carnitine, TMA and methionine. After adjustment for age, sex, eGFR and uACR, the association with all-cause death remained significant for betaine (HR = 1.18, [1.08; 1.30], p = 0.0004) and homocysteine (HR = 1.18, [1.09; 1.27], p < 0.0001). In the fully adjusted models, only homocysteine remained significant (HR = 1.16, [1.06; 1.27], p = 0.0011). For HFrH and/or CV death, no association with the biomarkers remained significant in the fully adjusted models.

In an exploratory analysis, we analyzed whether the relationship between nutritional biomarkers and outcomes was modified when stratifying on relevant sub-groups (Additional file 2: Fig. S3A–C). Interestingly, the association between nutritional biomarkers of red-meat and vegetable intakes with clinical outcomes was not strongly influenced by history of CAD, obesity (BMI ≥ 30 kg/m^2^) and possible history of HF (NT-propBNP ≥ 125 pg/mL).

## Discussion

In this monocentric cohort of patients with T2D, we were able to establish that nutritional biomarkers of red meat consumption (methylamines, TMAO and related compounds) and of vegetables (thio-amino-acids, homocysteine and related compounds) were associated with incident acute HFrH, HFrH and/or CV death, and all-cause death, in univariate analysis. However, when performing adjustment for renal parameters, betaine was the only biomarker remaining associated with incident HFrH. When adjusting on cardiac biomarkers (NT-proBNP and history of CAD), we found no remaining effect of those nutritional biomarkers. Likewise, no biomarkers were significantly associated with HFrH and/or CV death in the fully adjusted models. Interestingly, we found that homocysteine concentration was a significant and independent risk factors for all-cause death after multiple adjustments including renal and cardiac biomarkers, and after a conservative correction for multiple testing.

### Characteristics of patients with HF—external validation

In this study, we confirmed that established risk factors for HFrH were present such as older age, longer diabetes duration, increased systolic blood pressure (SBP) and NT-proBNP concentrations, and renal parameters (CKD and macroalbuminuria). Of note, CAD was found in approximately half of the participants with incident HFrH, in agreement with the data from large cohorts studying this condition [16]. SURDIAGENE cohort is of peculiar value as it is specific of T2D and its related complications. We namely found that those participants who had HFrH during the follow-up had higher renal and retinal complications compared to those who remained free of HFrH during follow-up. This point was previously suggested in the EMPAREG Outcome study where the greater risk of HF was significantly associated with a greater number of microvascular complications [17]. Particularly, we found a greater risk of HFrH in those participants with diabetic retinopathy, in accordance with previous reports on microvascular disease and HFrH [18, 19].

### Dietary approach to prevent HF

The current approach considered nutritional biomarkers potentially indicative of red meat (TMAO, betaine, choline and carnitine) and of vegetable consumption, more specifically folate intake (homocysteine, cysteine and methionine). An intake of red meat (specifically ≤ 28 g/day of pork, lamb and beef) and of vegetables ≥ 200 g/day is part of the EAT-Lancet score. So far, to our knowledge, the EAT-Lancet score was not reported in the field of HF. Red (and processed) meat consumption and cardiovascular disease was reviewed by Ferreira et al. and altogether evidenced a deleterious effect of red meat consumption, while plant protein intakes were seen very positively. [20] However, no specific mention of HF was available to our knowledge.

The DASH (Dietary Approach to Stop Hypertension) emphasizes a diet focusing on decreased intake of red meat and increased intake of vegetables, compared to the usual American diet [21]. Of interest, the DASH diet was largely tested and provided rather positive effects in the context of established HF, with the meta-analysis of two studies concluding to a HR of HF at 0.71 suggesting a significant benefit of this dietary approach, in an analysis in which subjects with T2D were excluded [22]. However, the DASH diet is also associated with a restriction in salt intake, which limits the conclusion to a specific effect of this diet on HF.

The Mediterranean diet including the standardized PREDIMED diet also insists on the reduction of the red meat intake and the increase in vegetables consumption compared to usual diet [23]. Of interest, the Mediterranean diet was associated with a reduction in HF incidence and mortality in cohort studies. Conversely, using a randomized controlled strategy, the PREDIMED trial found no significant effect of the evaluated nutritional interventions on incident HF. The study population included 7403 participants without established HF, also including 3610 with T2D. However, the incidence of HF was rather low with a total of 94 events during a follow-up of 4.8 years. In the HOPE-2 trial, homocysteine lowering with folate was not associated with differences in hospitalization for HF [24]. In addition, a Cochrane meta-analysis suggested no role of vitamin B-based homocysteine-lowering interventions on all-cause death [25].

### Methylamines and health outcomes

To our knowledge, the association between TMAO and related metabolites and incident HFrH was poorly examined in the literature. The majority focused on populations with established HF, which was summarized in a recent meta-analysis, showing that TMAO was a risk factor for MACE and all-cause death [26]. We previously found an association between plasma TMAO concentrations and MACE/death in the SURDIAGENE cohort, which was not restricted to patients with established HF [7]. The results presented here, considering multivariable models, did not reach statistical significance regarding an impact of TMAO on risk of HF in persons living with T2D. Of interest, the plasma concentration of TMAO was reported to be higher in those patients with HF compared to the others [27, 28]. This could partly explain our finding of an association between TMAO and HFrH, which did not persist when adjusting for other cardiac biomarkers, even if plasma concentrations of TMAO and linked metabolites were not correlated to NT-proBNP concentrations. Introducing data on gut microbiota, which is clearly a key explanation when studying this metabolite, is well-above the scope of this paper but should be addressed in future studies [26].

### Thio-amino-acids and health outcomes

Our results suggest no obvious and strong relationship between thio-amino-acids and HFrH. Particularly, homocysteine was associated with the HFrH in univariate model but this relationship was not sustained when renal and/or cardiac biomarkers were added to the models. Very similar results were found in patients from the IDNT trial, with T2D and overt nephropathy [29]. However, we found that homocysteine concentration was associated with all-cause death, even when adjusting on age, sex, and, notably, renal and cardiac biomarkers. Of interest, a recent meta-analysis found that homocysteine concentrations were higher in patients with HF compared with those without [30]. Our results suggest against a strong effect of folate consumption regarding HF. However, it can also be argued that vegetables are part of a dietary pattern which proved to be beneficial regarding all-cause death in the PREDIMED trial [31]. This clearly illustrates the complexity of nutritional intervention and the difficulty to isolate the effect of one specific nutrient.

The interpretation of our findings regarding homocysteine association with all-cause death remains an open question. Our data were observational, while interventions altogether tend to be negative. This implies that we do not have enough scientific evidence to suggest for an increase in nutrients able to decrease homocysteine to lower all-cause death. However, our results can also be viewed as an important finding to establish biomarkers associated with key outcomes, in terms of epidemiology. In a computerized era, whether inclusion of homocysteine will lead to a better classification of HFrH-hazard will require continuous efforts, but our results are a strong impetus for such a step on the way to 4P medicine.

### Limitations and strengths

The current study has limitations to acknowledge. The primary outcome considered in the current analysis can be questioned. Indeed, in HF trials, a composite endpoint combining CV death and hospitalization for HF is very consistently used. However, as the majority of the SURDIAGENE cohort was not affected by HF at baseline, we focused on HFrH, defined as the first occurrence of acute HF leading to hospitalization and/or death. CV death was not specific enough at variance with HF trials where CV death is mostly secondary to the ominous evolution of the condition. Still, the study of the composite event of HFrH and/or CV death was given along with the main outcome. One obvious question in our present approach was whether the lack of strong effect was related to a weak statistical power. After multiple adjustment, we found that the upper limit of the 95% confidence interval of the HR was 1.27 for betaine and 1.21 for homocysteine (for an increase of 1 SD of the given parameter), which does not support a strong relationship between these proxies of nutritional intakes and incident heart failure. So, even if we must acknowledge for a limited statistical power, considering a larger population and a greater number of events is unlikely to lead to highly clinically relevant association of red meat and folates intakes with HF. Also, the deleterious effect of homocysteine on all-cause death can be challenged. Whether this is due to a spurious result secondary to multiple testing was examined. When applying a very conservative Bonferroni correction (24 tests), homocysteine was still associated with all-cause death, suggesting that it truly represents a relevant risk factor for death in patients with T2D. Moreover, the analyses presented here relied on previously established observations of the link between TMAO and derivates and red meat intake, on one hand, and between homocysteine and folate intake, on the other hand. Unfortunately, this study could not confront nutritional biomarkers to individual nutritional habits. Also, the exposure (dosing of nutritional biomarkers) was measured only once, at baseline. Also, the exposure (dosing of nutritional biomarkers) was measured only once, at baseline. Therefore, the study results are based on the hypothesis that these determinations were representative of the mean values of the biomarkers. Repeated data would be needed to increase the study accuracy. Thirdly, the observational design of our study leads to low-grade guidelines, even though randomized clinical trials might be challenging requiring some comparisons between animal meat and plant-derived meat, as mentioned by Ferreira et al*.* [20]. Lastly, a history of HF prior to inclusion in the cohort was not established. No questionnaire is available, to our best knowledge, to establish chronic or previous HF, in a similar fashion as the Rose questionnaire for CAD. However, when we stratified on the recommended threshold of NT-proBNP to indicate potential HF (≥ 125 pg/mL) [32], we did not evidence a strong difference regarding the association of TMAO with HFrH between the two groups. Of note, caution must be taken as our subgroup analysis was not pre-specified.

This study also has some strengths including its long-term follow-up, the adjudication of clinical endpoints by an independent adjudication committee and the use of state of the art methodological determinations using mass spectrometry showing good stability and reproducibility.

## Conclusion

To summarize, our study searched for an association between methylamines but also between thio-amino-acid plasma concentrations and severe HF. We did not evidence any major effect of these nutritional biomarkers associated with red meat consumption and folate intakes on incident HF in patients with T2D. The research strategy applied here remains rarely used while it could be considered as an alternative to time- and resource-consuming food questionnaire, to establish the impact of nutritional environment on health outcomes, such as HF. Our results could prove interesting with regard to other specific nutritional biomarkers for the prevention of HF. In addition, multiple approaches adding nutritional biomarkers to other exposome markers are surely a relevant research strategy in complex conditions such as cardiovascular metabolic diseases.

## Supplementary Information

**Additional file 1. **Biological determinations. Nutritional BM and HFrH in T2D. Details of the quantification of methylamines and amino-acids

**Additional file 2. **Nutritional BM and HFrH in T2D. Supplemental Figures and Tables.

**Additional file 3.** SURDIAGENE ORGANISATION (Committees and staff).

## Data Availability

The French regulatory authorities do not allow sharing of individual health data which can lead to patients’ reidentification. Therefore, the entire dataset supporting the conclusions of this article cannot be made publicly available. However, specific subsets of the data may be provided by the corresponding author, on reasonable request.

## References

[1] IDF Atlas 9th edition and other resources [Internet]. https://diabetesatlas.org/en/resources/. Accessed 30 Apr 2021.

[2] Rawshani A, Rawshani A, Franzén S, Eliasson B, Svensson A-M, Miftaraj M (2017). Mortality and cardiovascular disease in type 1 and type 2 diabetes. N Engl J Med.

[3] Rawshani A, Rawshani A, Franzén S, Sattar N, Eliasson B, Svensson A-M (2018). Risk factors, mortality, and cardiovascular outcomes in patients with type 2 diabetes. N Engl J Med.

[4] Ohkuma T, Komorita Y, Peters SAE, Woodward M (2019). Diabetes as a risk factor for heart failure in women and men: a systematic review and meta-analysis of 47 cohorts including 12 million individuals. Diabetologia.

[5] Jenkins DJA, Dehghan M, Mente A, Bangdiwala SI, Rangarajan S, Srichaikul K (2021). Glycemic Index, Glycemic Load, and Cardiovascular Disease and Mortality. N Engl J Med.

[6] Ponikowski P, Voors AA, Anker SD, Bueno H, Cleland JGF, Coats AJS, et al. 2016 ESC Guidelines for the diagnosis and treatment of acute and chronic heart failure: The Task Force for the diagnosis and treatment of acute and chronic heart failure of the European Society of Cardiology (ESC). Developed with the special contribution of the Heart Failure Association (HFA) of the ESC. Eur J Heart Fail. 2016;18:891–975. 10.1002/ejhf.592 27207191

[7] Croyal M, Saulnier P-J, Aguesse A, Gand E, Ragot S, Roussel R, et al. Plasma Trimethylamine N-Oxide and Risk of Cardiovascular Events in Patients With Type 2 Diabetes. J Clin Endocrinol Metab. 2020;105. 10.1210/clinem/dgaa188 32301490

[8] Willett W, Rockström J, Loken B, Springmann M, Lang T, Vermeulen S (2019). Food in the Anthropocene: the EAT-Lancet Commission on healthy diets from sustainable food systems. Lancet Lond Engl.

[9] Hadjadj S, Fumeron F, Roussel R, Saulnier P-J, Gallois Y, Ankotche A (2008). Prognostic value of the insertion/deletion polymorphism of the ACE gene in type 2 diabetic subjects: results from the Non-insulin-dependent Diabetes, Hypertension, Microalbuminuria or Proteinuria, Cardiovascular Events, and Ramipril (DIABHYCAR), Diabete de type 2, Nephropathie et Genetique (DIAB2NEPHROGENE), and Survie, Diabete de type 2 et Genetique (SURDIAGENE) studies. Diabetes Care.

[10] Levey AS, Stevens LA, Schmid CH, Zhang YL, Castro AF, Feldman HI (2009). A new equation to estimate glomerular filtration rate. Ann Intern Med.

[11] Austin PC, Lee DS, Fine JP (2016). Introduction to the analysis of survival data in the presence of competing risks. Circulation.

[12] Fine JP, Gray RJ (1999). A proportional hazards model for the subdistribution of a competing risk. J Am Stat Assoc.

[13] Sauerbrei W, Meier-Hirmer C, Benner A, Royston P (2006). Multivariable regression model building by using fractional polynomials: description of SAS, STATA and R programs. Comput Stat Data Anal.

[14] R Core Team. R: A Language and Environment for Statistical Computing [Internet]. Vienna, Austria: R Foundation for Statistical Computing; 2020. https://www.R-project.org/

[15] Gray B. cmprsk: Subdistribution Analysis of Competing Risks [Internet]. 2020 [cited 2021 Jan 1].: https://CRAN.R-project.org/package=cmprsk

[16] Low Wang CC, Hess CN, Hiatt WR, Goldfine AB (2016). Clinical update: cardiovascular disease in diabetes mellitus: atherosclerotic cardiovascular disease and heart failure in type 2 diabetes mellitus—mechanisms, management, and clinical considerations. Circulation.

[17] Verma S, Wanner C, Zwiener I, Ofstad AP, George JT, Fitchett D (2019). Influence of microvascular disease on cardiovascular events in type 2 diabetes. J Am Coll Cardiol.

[18] Tromp J, Lim SL, Tay WT, Teng T-HK, Chandramouli C, Ouwerkerk W (2019). Microvascular disease in patients with diabetes with heart failure and reduced ejection versus preserved ejection fraction. Diabetes Care.

[19] Sandesara PB, O’Neal WT, Kelli HM, Samman-Tahhan A, Hammadah M, Quyyumi AA (2018). The prognostic significance of diabetes and microvascular complications in patients with heart failure with preserved ejection fraction. Diabetes Care.

[20] Ferreira JP, Sharma A, Zannad F (2021). The future of meat: health impact assessment with randomized evidence. Am J Med.

[21] Sacks FM, Svetkey LP, Vollmer WM, Appel LJ, Bray GA, Harsha D (2001). Effects on blood pressure of reduced dietary sodium and the Dietary Approaches to Stop Hypertension (DASH) diet. DASH-Sodium Collaborative Research Group. N Engl J Med.

[22] Salehi-Abargouei A, Maghsoudi Z, Shirani F, Azadbakht L (2013). Effects of Dietary Approaches to Stop Hypertension (DASH)-style diet on fatal or nonfatal cardiovascular diseases–incidence: a systematic review and meta-analysis on observational prospective studies. Nutr Burbank Los Angel Cty Calif.

[23] Estruch R, Ros E, Salas-Salvadó J, Covas M-I, Corella D, Arós F (2018). primary prevention of cardiovascular disease with a Mediterranean diet supplemented with extra-virgin olive oil or nuts. N Engl J Med.

[24] Lonn E, Yusuf S, Arnold MJ, Sheridan P, Pogue J, Micks M (2006). Homocysteine lowering with folic acid and B vitamins in vascular disease. N Engl J Med.

[25] Martí-Carvajal AJ, Solà I, Lathyris D (2015). Homocysteine-lowering interventions for preventing cardiovascular events. Cochrane Database Syst Rev.

[26] Li W, Huang A, Zhu H, Liu X, Huang X, Huang Y (2020). Gut microbiota-derived trimethylamine N-oxide is associated with poor prognosis in patients with heart failure. Med J Aust.

[27] Trøseid M, Ueland T, Hov JR, Svardal A, Gregersen I, Dahl CP (2015). Microbiota-dependent metabolite trimethylamine-N-oxide is associated with disease severity and survival of patients with chronic heart failure. J Intern Med.

[28] Tang WHW, Wang Z, Fan Y, Levison B, Hazen JE, Donahue LM (2014). Prognostic value of elevated levels of intestinal microbe-generated metabolite trimethylamine-N-oxide in patients with heart failure: refining the gut hypothesis. J Am Coll Cardiol.

[29] Friedman AN, Hunsicker LG, Selhub J, Bostom AG, Collaborative Study Group (2005). Total plasma homocysteine and arteriosclerotic outcomes in type 2 diabetes with nephropathy. J Am Soc Nephrol JASN..

[30] Jin N, Huang L, Hong J, Zhao X, Chen Y, Hu J (2021). Elevated homocysteine levels in patients with heart failure: a systematic review and meta-analysis. Medicine.

[31] Martínez-González MA, Sánchez-Tainta A, Corella D, Salas-Salvadó J, Ros E, Arós F (2014). A provegetarian food pattern and reduction in total mortality in the Prevención con Dieta Mediterránea (PREDIMED) study. Am J Clin Nutr.

[32] McDonagh TA, Metra M, Adamo M, Gardner RS, Baumbach A, Böhm M (2021). 2021 ESC Guidelines for the diagnosis and treatment of acute and chronic heart failure. Eur Heart J.

