# Supplementary material for: Nutritional biomarkers and heart failure requiring hospitalization in patients with type 2 diabetes: the SURDIAGENE cohort
Source: Cardiovasc Diabetol. 2022 Jun 9;21:101. doi: 10.1186/s12933-022-01505-9 (PMC9185908; doi:10.1186/s12933-022-01505-9)
Supplement: Supplementary file 1 — Additional file 1. Biological determinations. Nutritional BM and HFrH in T2D. Details of the quantification of methylamines and amino-acids [file 12933_2022_1505_MOESM1_ESM.docx]

**ADDITIONAL FILE 1: BIOLOGICAL DETERMINATION**

**Nutritional biomarkers and heart failure requiring hospitalization in patients with type 2 diabetes - the SURDIAGENE cohort.**

Matthieu Wargny, Mikaël Croyal, Stéphanie Ragot, Elise Gand, David Jacobi, Jean-Noël Trochu, Xavier Prieur, Cédric Le May, Thomas Goronflot, Bertrand Cariou, Pierre-Jean Saulnier, Samy Hadjadj for the SURDIAGENE study group

**Quantification of methylamines –** Trimethylamine N-oxide (TMAO), trimethylamine (TMA), betaine, choline, and carnitine concentrations were determined by liquid chromatography-tandem mass spectrometry (LC-MS/MS). All solvents used were LC-MS grade and purchased from Biosolve (Valkenswaard, Netherlands). Standard compounds were obtained from Sigma Aldrich (Saint-Quentin Fallavier, France). A pool of reference standard solutions was prepared and serially diluted in acetonitrile to obtain seven standard solutions ranging from 0.05 to 100 µmol/L. Exogenous internal standards (10 µL) diluted at 25 µmol/L in acetonitrile (^2^H_9_-choline, ^2^H_9_-carnitine, ^13^C_2_-betaine, [^13^C_3_,^15^N]-TMA and ^2^H_9_-TMAO) were added to 20 µL of standard solutions and plasma samples. All samples were then treated with 75 µL of tert-butyl-bromoacetate (TMA derivatization) diluted at 50 mmol/L in acetonitrile and 10 µL of 70% ammonium hydroxide solution before mixing and incubation in the dark, at room temperature, for 30 min. Then, 50 µL of acetonitrile containing 1% formic acid were added and samples were centrifuged for 10 min at 10,000× g (20°C). Supernatants were then transferred to vials for LC-MS/MS analyses, performed on a Xevo^®^ TQD mass spectrometer with an electrospray interface and an Acquity H-Class^®^ UPLC^TM^ device (Waters Corporation, Milford, MA, USA). Samples (5 µL) were injected onto an HILIC-BEH column (1.7 µm, 2.1 × 100 mm, Waters Corporation) held at 35 °C. Compounds were separated using a linear gradient of mobile phase B (98% acetonitrile, 0.1% formic acid) in mobile phase A (10 mmol/L ammonium acetate, 0.1% formic acid) at a flow rate of 400 µL/min. Mobile phase A was kept constant for 1 min at 1%, linearly increased from 1% to 45% for 6.5 min, kept constant for 1 min, returned to the initial condition over 1 min, and kept constant for 1.5 min before the next injection. Targeted compounds were then detected by the mass spectrometer with the electrospray interface operating in the positive ion mode (capillary voltage, 1.5 kV; desolvatation gas (N_2_) flow and temperature, 650 L/h and 350°C; source temperature, 150 °C). The multiple reaction monitoring mode was applied for MS/MS detection as detailed below. Chromatographic peak area ratios between unlabeled compounds and their respective internal standards constituted the detector responses. Standard solutions were used to plot calibration curves for quantification. The linearity was expressed by the mean R² which was greater than 0.998 for all compounds (linear regression, 1/x weighting, origin excluded).

**Quantification of amino-acids –** Cysteine, homocysteine and methionine plasma concentrations were performed by LC-MS/MS on a Xevo^®^ Triple-Quadrupole mass spectrometer with an electrospray ionization interface equipped with an Acquity H-Class^®^ UPLC^TM^ device (Waters Corporation, Milford, MA, USA). All solvents used were LC-MS grade and purchased from Biosolve. Standard compounds were obtained from Sigma Aldrich. Individual stock solutions (10 mmol/L) of amino-acid and ^2^H_3_-cysteine were prepared in 0.1 M HCl. A pool of unlabeled standard solutions was prepared and serially diluted in water to obtain seven standard solutions ranging from 0.1 to 50 µmol/L. A pool solution of labeled ^2^H_3_-cysteine and [^13^C,^2^H_3_]-methionine (50 µmol/L), was prepared in water. The standard solutions and serum samples (20 µL) were then extracted with 100 µL of methanol and 50 µL of the ^2^H_3_-cysteine solution. The samples were mixed and centrifuged at 10 000 × *g* and 10 °C for 15 min to remove the precipitated proteins. The supernatants were collected and dried under a gentle stream of nitrogen (45 °C). The derivatization step was performed by dissolving the dried extract in 100 µL of a freshly prepared butanol solution containing 5% acetyl chloride and kept at 60 °C for 30 min. The solvent was then removed under a gentle stream of nitrogen (60 °C). The dried samples were dissolved in 100 µL of water containing 0.1% formic acid and 50 µmol/L TCEP and injected into the LC-MS/MS system. Samples (10 µL) were injected onto an Acquity BEH-C_18_ column (1.7 µm; 2.1 × 100 mm, Waters Corporation) held at 60 °C, and compounds were separated with a linear gradient of mobile phase B (0.1% formic acid in methanol) in mobile phase A (0.1% formic acid in water) at a flow rate of 400 µL/min. Mobile phase B was kept constant at 1% for 0.5 min, linearly increased from 1% to 95% for 4.5 min, kept constant for 1 min, returned to the initial condition over 0.5 min, and kept constant for 1.5 min before the next injection. Target compounds were then detected by the mass spectrometer with the electrospray interface operating in the positive ion mode (capillary voltage, 3 kV; desolvatation gas (N_2_) flow, 650 L/h; desolvatation gas temperature, 350 °C; source temperature, 120 °C). The multiple reaction monitoring mode was applied for MS/MS detection as detailed below. Chromatographic peak area ratios between unlabeled compounds and ^2^H_3_-cysteine (cysteine and homocysteine) and [^13^C,^2^H_3_]-methionine (methionine) constituted the detector responses. Standard solutions were used to plot the calibration curves for quantification. The assay linearity was expressed by the mean *R*², which was greater than 0.994 for all compounds (linear regression, 1/x weighting, origin excluded).

**Multiple reaction monitoring (MRM) transitions used for LC-MS/MS detection.**

| **Compound** | **MRM transition (*m/z*)** | **Cone/collision (V)** |
| --- | --- | --- |
| TMAO | 75.9 → 58.9 | 20/11 |
| ^2^H_9_-TMAO | 85.0 → 68.0 | 20/11 |
| TMA | 174.1 → 118.0 | 35/18 |
| [^13^C_3_, ^15^N]-TMA | 178.1 → 122.0 | 35/18 |
| Betaine | 118.1 → 58.1 | 40/22 |
| ^13^C_2_-betaine | 120.1 → 58.1 | 40/22 |
| Choline | 104.1 → 60.1 | 40/15 |
| ^2^H_9_-choline | 113.2 → 69.1 | 40/15 |
| Carnitine | 162.1 → 103.0 | 25/14 |
| ^2^H_9_-carnitine | 171.1 → 112.0 | 25/14 |
| Cysteine | 178.1 → 75.9 | 30/15 |
| Homocysteine | 192.1 → 89.9 | 30/15 |
| ^2^H_3_-cysteine | 181.1 → 78.9 | 30/15 |
| Methionine | 206.2 → 103.9 | 30/15 |
| [^13^C,^2^H_3_]-methionine | 210.2 → 107.9 | 30/15 |
