# Supplementary material for: Nutritional biomarkers and heart failure requiring hospitalization in patients with type 2 diabetes: the SURDIAGENE cohort
Source: Cardiovasc Diabetol. 2022 Jun 9;21:101. doi: 10.1186/s12933-022-01505-9 (PMC9185908; doi:10.1186/s12933-022-01505-9)
Supplement: Supplementary file 2 — Additional file 2. Nutritional BM and HFrH in T2D. Supplemental Figures and Tables. [file 12933_2022_1505_MOESM2_ESM.docx]

**ADDITIONAL FILE 2: SUPPLEMENTAL FIGURES AND TABLES**

**Nutritional biomarkers and heart failure requiring hospitalization in patients with type 2 diabetes - the SURDIAGENE cohort.**

Matthieu Wargny, Mikaël Croyal, Stéphanie Ragot, Elise Gand, David Jacobi, Jean-Noël Trochu, Xavier Prieur, Cédric Le May, Thomas Goronflot, Bertrand Cariou, Pierre-Jean Saulnier, Samy Hadjadj for the SURDIAGENE study group

**Figure S1. Pairwise complete correlations plot for clinical and biological parameters
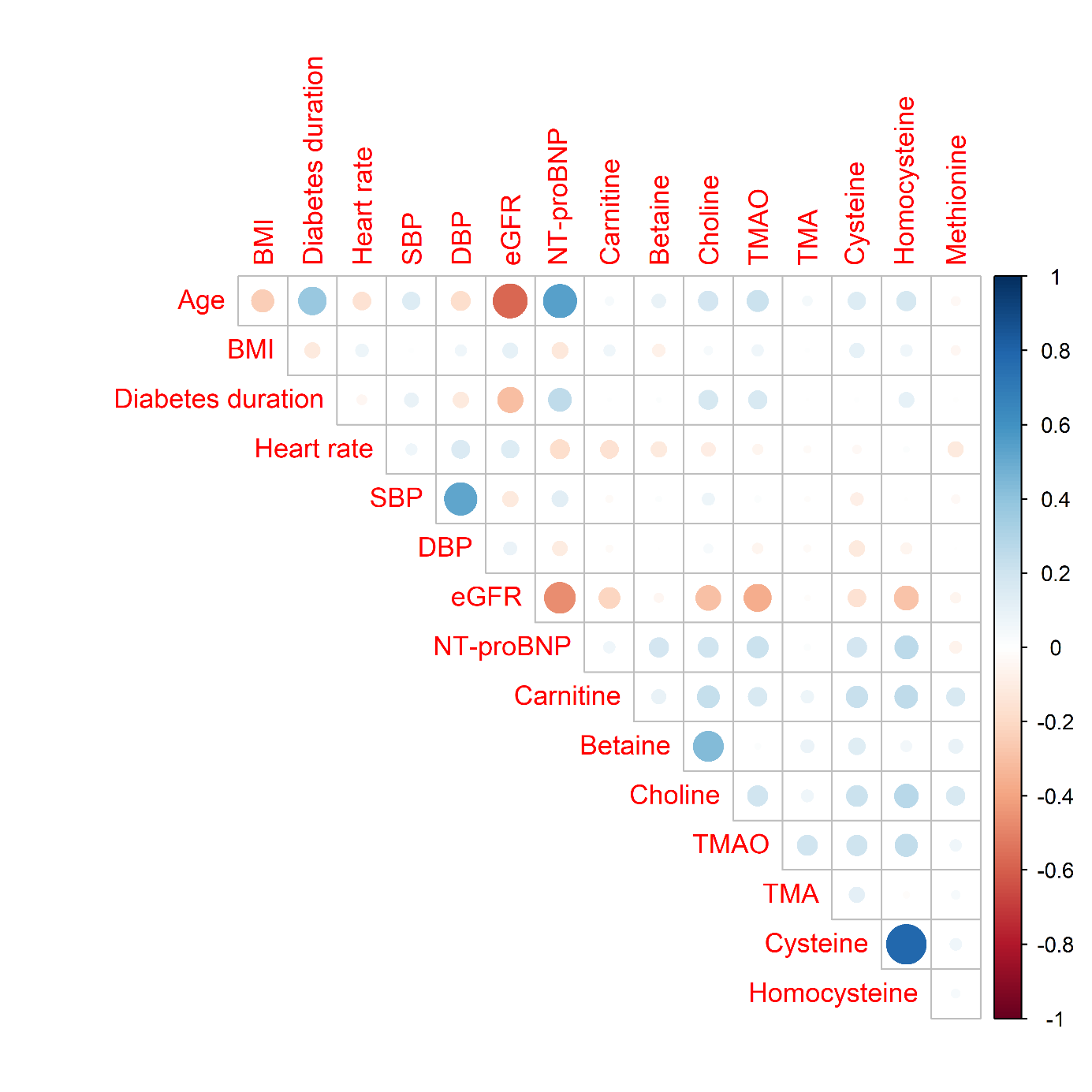
**

Spearman’s rho correlation coefficients are computed, using all complete pairs of observations for a given pair of variables.

**Abbreviations:** BMI: body mass index; DBP: diastolic blood pressure; eGFR: estimated glomerular filtration rate calculated with the CKD-EPI 2009-formula; NT-proBNP: N-terminal prohormone of brain natriuretic peptide; SBP: systolic blood pressure; TMA: trimethylamine; TMAO: trimethylamine N-oxide

**Figure S2. Cumulative Incidence Function for all-cause death**

**
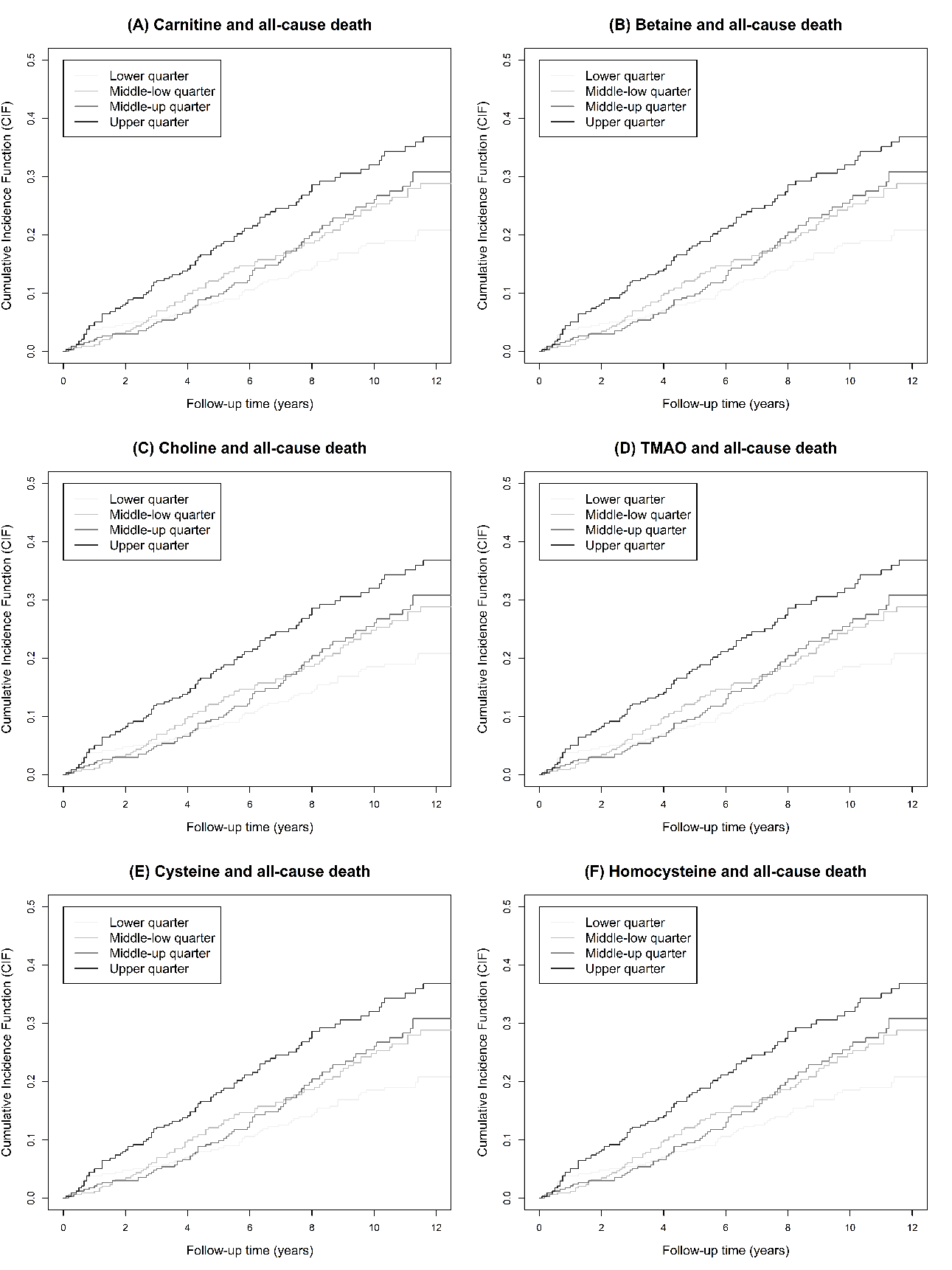
**

Quartile values for the different parameters of interest: carnitine (median = 42.6, [25^th^-75^th^] percentile = [35.4-50.7]); betaine (31.5, [24.6-38.9]); choline (1.43, [1.22-1.68]); TMAO (6.8, [4.2-12.8]); cysteine (23, [13-39]); homocysteine (8.9, [4.7-14.8])

Abbreviations: TMAO: trimethylamine N-oxide.

**Fig S3.A Survival analysis for HFrH and all-cause death stratified by CAD status**


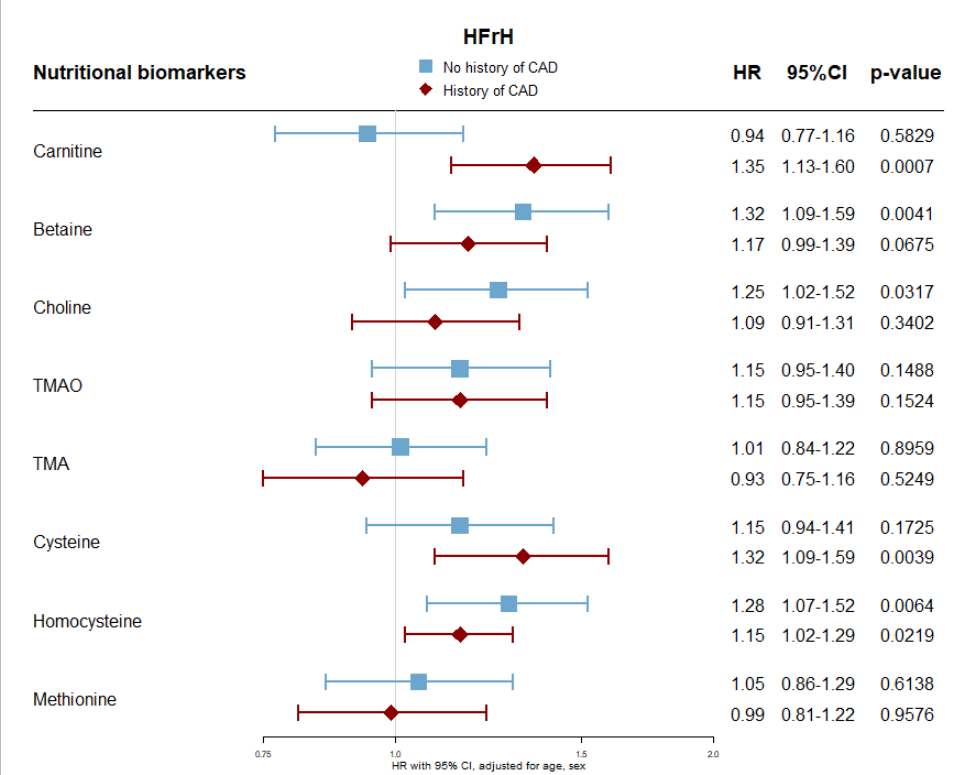

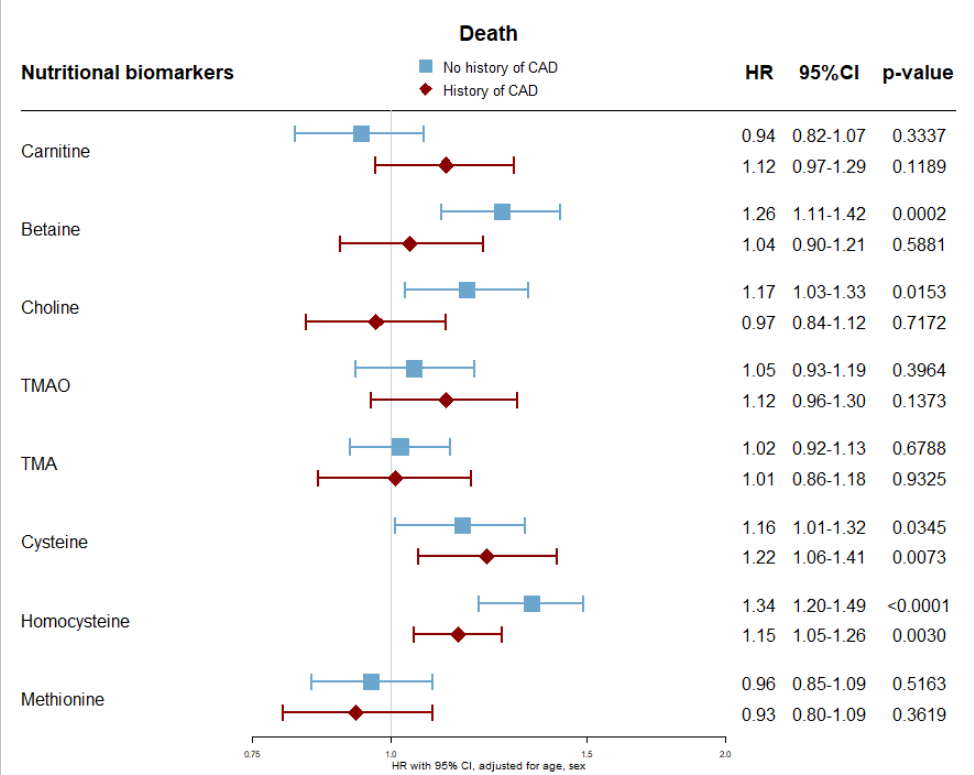


Cause-specific hazard models adjusted for age and sex. All HR are given per 1 SD of the given parameter. TMAO was natural-log transformed before standardization. The nutritional biomarkers were tested separately from each other in the different models.

Abbreviations: CAD: coronary artery disease; HFrH: Heart Failure requiring Hospitalization, defined as the first occurrence of acute HF leading to hospitalization and/or death; HR: Hazard-ratio; TMA: trimethylamine; TMAO: trimethylamine N-oxide

**Fig S3.B Survival analysis for HFrH and all-cause death stratified by obesity status**


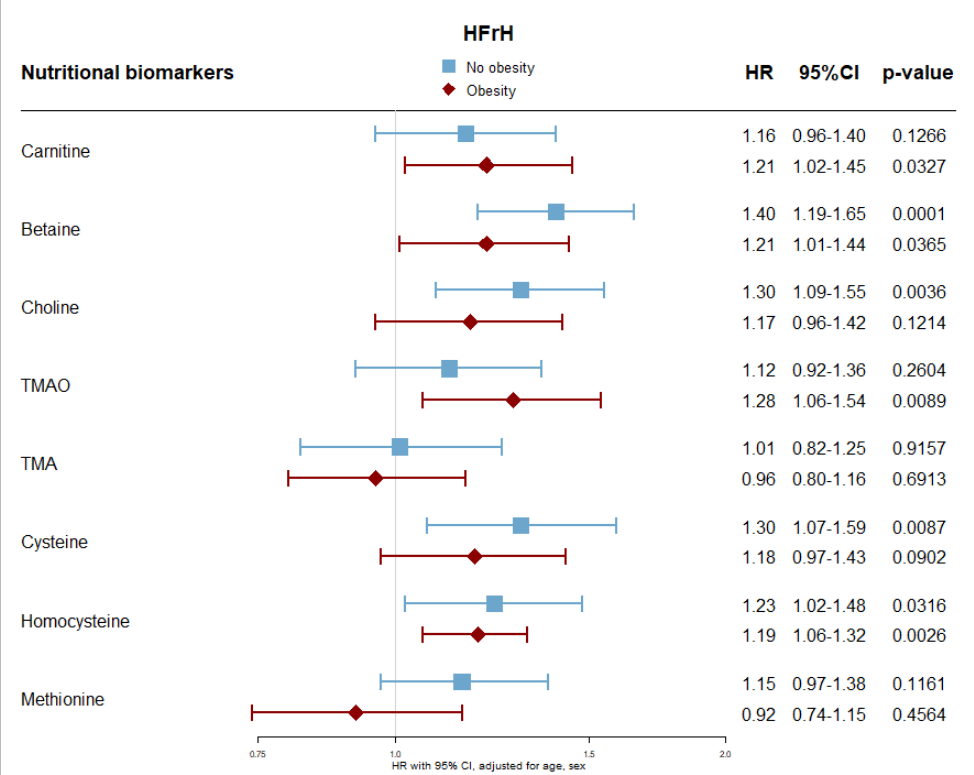

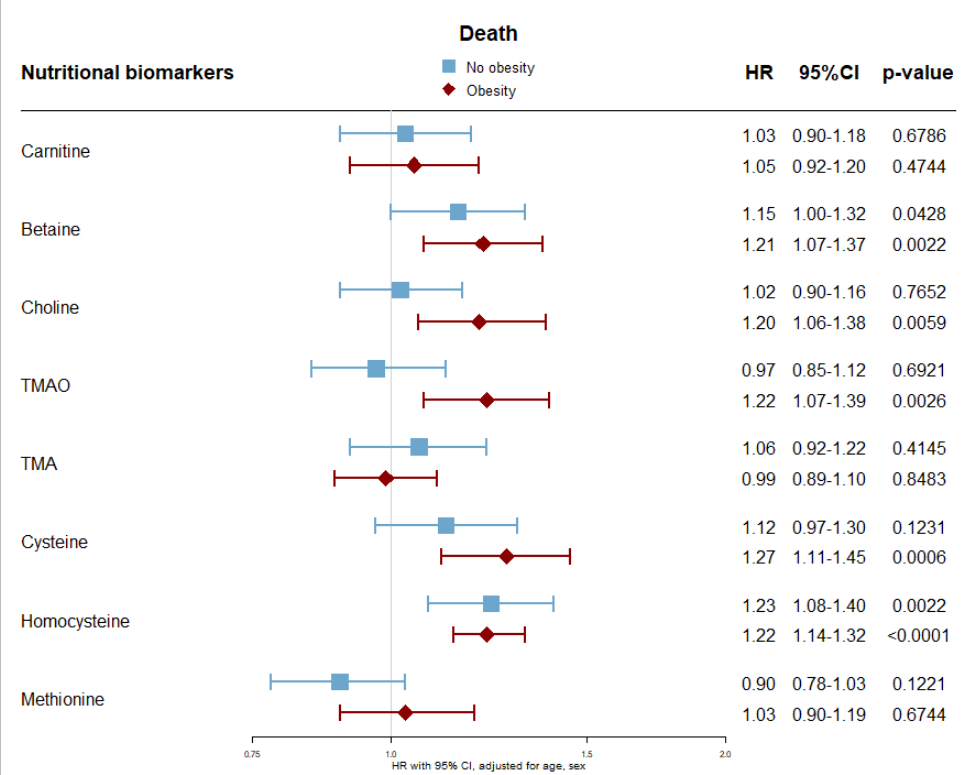


Cause-specific hazard models adjusted for age and sex. All HR are given per 1 SD of the given parameter. TMAO was natural-log transformed before standardization. The nutritional biomarkers were tested separately from each other in the different models.

Abbreviations: HFrH: Heart Failure requiring Hospitalization, defined as the first occurrence of acute HF leading to hospitalization and/or death; HR: Hazard-ratio; TMA: trimethylamine; TMAO: trimethylamine N-oxide

**Fig S3.C Survival analysis for HFrH and all-cause death stratified by NT-proBNP value**


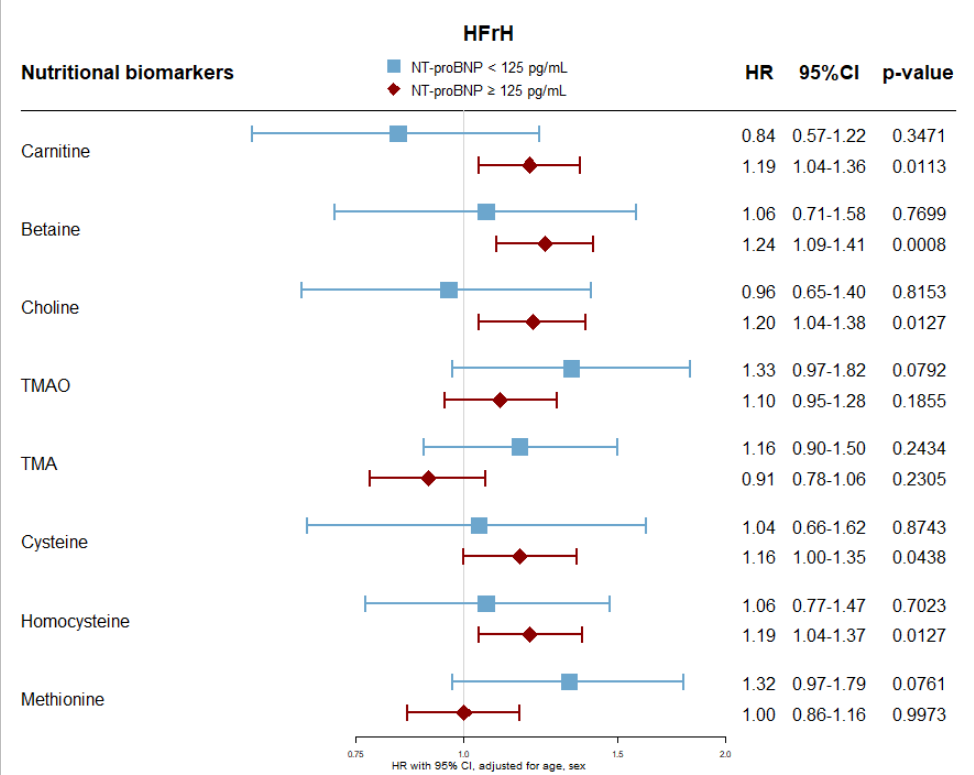

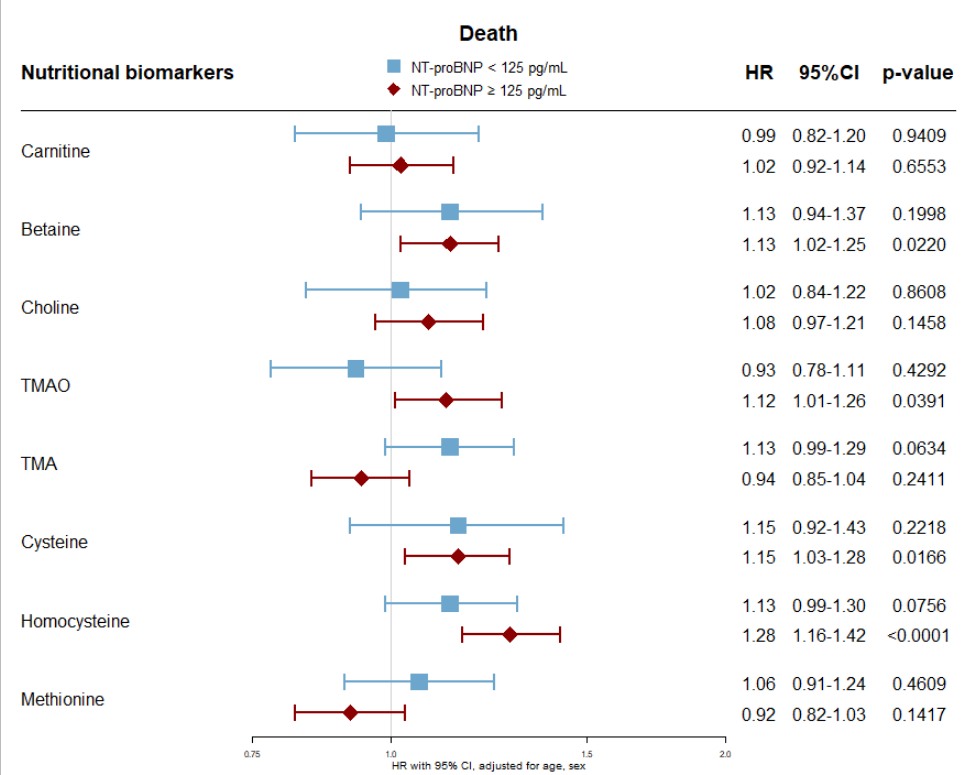


Cause-specific hazard models adjusted for age and sex. All HR are given per 1 SD of the given parameter. TMAO was natural-log transformed before standardization. The nutritional biomarkers were tested separately from each other in the different models.

Abbreviations: HFrH: Heart Failure requiring Hospitalization, defined as the first occurrence of acute HF leading to hospitalization and/or death; HR: Hazard-ratio; TMA: trimethylamine; TMAO: trimethylamine N-oxide

**Table S1. Survival analysis for HFrH – relative incidences (subdistribution hazard models)**

|  | **M_1_** | | **M_2_** | | **M_3A_** | | **M_3B_** | | **M_4_** | |
| --- | --- | --- | --- | --- | --- | --- | --- | --- | --- | --- |
|  | **HR (95%CI)** | ***P*-value** | **HR (95%CI)** | ***P*-value** | **HR (95%CI)** | ***P*-value** | **HR (95%CI)** | ***P*-value** | **HR (95%CI)** | ***P*-value** |
| **Methylamines** |  |  |  |  |  |  |  |  |  |  |
| Carnitine | 1.20 [1.03; 1.40] | 0.019 | 1.19 [1.02; 1.38] | 0.025 | 1.14 [0.99; 1.31] | 0.075 | 1.13 [0.97; 1.32] | 0.11 | 1.14 [0.98; 1.32] | 0.085 |
| Betaine | 1.25 [1.13; 1.39] | <0.0001 | 1.22 [1.09; 1.37] | 0.0007 | 1.10 [0.96; 1.25] | 0.18 | 1.24 [1.10; 1.40] | 0.0004 | 1.10 [0.96; 1.27] | 0.16 |
| Choline | 1.29 [1.15; 1.45] | <0.0001 | 1.20 [1.06; 1.36] | 0.0036 | 1.00 [0.88; 1.13] | 1 | 1.08 [0.95; 1.23] | 0.26 | 0.97 [0.85; 1.11] | 0.70 |
| TMAO* | 1.28 [1.13; 1.44] | <0.0001 | 1.16 [1.01; 1.33] | 0.03 | 1.04 [0.90; 1.19] | 0.63 | 1.06 [0.91; 1.24] | 0.44 | 1.01 [0.86; 1.19] | 0.88 |
| TMA | 0.99 [0.86; 1.14] | 0.91 | 0.96 [0.83; 1.11] | 0.57 | 0.96 [0.82; 1.13] | 0.62 | 0.95 [0.82; 1.10] | 0.52 | 0.96 [0.82; 1.12] | 0.61 |
| **Thio-amino-acids** |  |  |  |  |  |  |  |  |  |  |
| Cysteine | 1.28 [1.12; 1.46] | 0.0002 | 1.15 [1.01; 1.31] | 0.036 | 1.01 [0.87; 1.18] | 0.87 | 1.07 [0.93; 1.23] | 0.32 | 1.00 [0.86; 1.17] | 0.96 |
| Homocysteine | 1.24 [1.12; 1.37] | <0.0001 | 1.16 [1.05; 1.28] | 0.0025 | 1.01 [0.88; 1.15] | 0.93 | 1.07 [0.95; 1.20] | 0.27 | 0.98 [0.84; 1.13] | 0.77 |
| Methionine | 1.01 [0.87; 1.19] | 0.86 | 1.05 [0.90; 1.23] | 0.51 | 1.08 [0.93; 1.25] | 0.32 | 1.08 [0.93; 1.25] | 0.31 | 1.09 [0.93; 1.27] | 0.28 |

*TMAO was natural-log transformed before standardization. All HR are given per 1 SD of the given parameter. M_1_: Model 1, univariate; M_2_: M_1_ covariates + age, sex; M_3A_: M_2_ covariates + history of CAD and log transformed NT-proBPNP; M_3B_: M_2_ covariates + eGFR and log transformed uACR; M_4_: M_3A_ covariates + eGFR and log transformed uACR. The nutritional biomarkers were tested separately from each other in the different adjustment models.

Abbreviations: CAD: coronary artery disease; eGFR: estimated glomerular filtration rate calculated with the CKD-EPI 2009-formula; HFrH: Heart Failure requiring Hospitalization, defined as the first occurrence of acute HF leading to hospitalization and/or death; HR: Hazard-ratio; NT-proBNP: N-terminal prohormone of brain natriuretic peptide; TMA: trimethylamine; TMAO: trimethylamine N-oxide; uACR: urine albumin/creatinine ratio

**Table S2 Survival analysis for HFrH, the composite HFrH and/or CV death event and all-cause death**

|  | **M_1_** | | **M_2_** | | **M_3A_** | | **M_3B_** | |
| --- | --- | --- | --- | --- | --- | --- | --- | --- |
| **Cause-specific HM for HFrH** | **HR (95_%CI_)** | ***P*-value** | **HR (95_%CI_)** | ***P*-value** | **HR (95_%CI_)** | ***P*-value** | **HR (95_%CI_)** | ***P*-value** |
| Carnitine | 1.20 [1.05; 1.37] | 0.0065 | 1.19 [1.04; 1.35] | 0.0090 | 1.13 [1.00; 1.29] | 0.052 | 1.13 [0.99; 1.29] | 0.073 |
| Betaine | 1.34 [1.20; 1.50] | <0.0001 | 1.30 [1.15; 1.47] | <0.0001 | 1.09 [0.96; 1.25] | 0.19 | 1.33 [1.17; 1.50] | <0.0001 |
| Choline | 1.35 [1.20; 1.52] | <0.0001 | 1.23 [1.08; 1.40] | 0.0016 | 0.98 [0.87; 1.12] | 0.79 | 1.09 [0.95; 1.25] | 0.21 |
| TMAO* | 1.32 [1.16; 1.50] | <0.0001 | 1.20 [1.05; 1.37] | 0.0073 | 1.12 [0.97; 1.28] | 0.12 | 1.10 [0.95; 1.27] | 0.20 |
| TMA | 1.01 [0.89; 1.15] | 0.86 | 0.99 [0.86; 1.13] | 0.88 | 0.97 [0.85; 1.12] | 0.73 | 0.98 [0.86; 1.12] | 0.79 |
| Cysteine | 1.38 [1.21; 1.58] | <0.0001 | 1.24 [1.08; 1.42] | 0.0022 | 1.12 [0.97; 1.30] | 0.13 | 1.13 [0.98; 1.31] | 0.092 |
| Homocysteine | 1.28 [1.17; 1.39] | <0.0001 | 1.20 [1.09; 1.32] | 0.0002 | 1.09 [0.96; 1.24] | 0.17 | 1.11 [0.98; 1.26] | 0.10 |
| Methionine | 1.02 [0.89; 1.18] | 0.73 | 1.05 [0.91; 1.20] | 0.54 | 1.04 [0.91; 1.20] | 0.53 | 1.08 [0.94; 1.24] | 0.26 |
| **Cause-specific HM for HFrH and/or CV death event** |  |  |  |  |  |  |  |  |
| Carnitine | 1.12 [1.01; 1.25] | 0.037 | 1.11 [1.00; 1.23] | 0.056 | 1.07 [0.96; 1.18] | 0.21 | 1.05 [0.95; 1.17] | 0.35 |
| Betaine | 1.27 [1.16; 1.40] | <0.0001 | 1.21 [1.09; 1.34] | 0.00029 | 1.03 [0.92; 1.15] | 0.62 | 1.23 [1.10; 1.36] | 0.00016 |
| Choline | 1.28 [1.17; 1.42] | <0.0001 | 1.16 [1.04; 1.29] | 0.0055 | 0.96 [0.86; 1.07] | 0.43 | 1.03 [0.92; 1.15] | 0.64 |
| TMAO* | 1.31 [1.19; 1.45] | <0.0001 | 1.20 [1.08; 1.34] | 0.00048 | 1.13 [1.01; 1.25] | 0.026 | 1.11 [0.99; 1.24] | 0.068 |
| TMA | 1.02 [0.93; 1.13] | 0.65 | 1.00 [0.90; 1.11] | 1 | 0.98 [0.88; 1.10] | 0.78 | 0.99 [0.90; 1.10] | 0.86 |
| Cysteine | 1.31 [1.17; 1.46] | <0.0001 | 1.18 [1.05; 1.32] | 0.0043 | 1.08 [0.96; 1.21] | 0.22 | 1.07 [0.95; 1.21] | 0.27 |
| Homocysteine | 1.28 [1.20; 1.37] | <0.0001 | 1.20 [1.12; 1.30] | <0.0001 | 1.13 [1.03; 1.25] | 0.013 | 1.12 [1.01; 1.23] | 0.024 |
| Methionine | 0.96 [0.85; 1.08] | 0.46 | 0.97 [0.87; 1.09] | 0.64 | 0.97 [0.87; 1.09] | 0.63 | 1.01 [0.91; 1.14] | 0.81 |
| **Cause-specific HM for all-cause death** |  |  |  |  |  |  |  |  |
| Carnitine | 1.05 [0.95; 1.15] | 0.32 | 1.04 [0.95; 1.15] | 0.37 | 1.01 [0.92; 1.11] | 0.80 | 1.01 [0.92; 1.11] | 0.87 |
| Betaine | 1.28 [1.18; 1.39] | <0.0001 | 1.18 [1.08; 1.29] | 0.0004 | 1.05 [0.96; 1.16] | 0.28 | 1.18 [1.08; 1.30] | 0.0004 |
| Choline | 1.26 [1.16; 1.38] | <0.0001 | 1.11 [1.01; 1.22] | 0.029 | 0.98 [0.89; 1.08] | 0.72 | 1.01 [0.91; 1.11] | 0.91 |
| TMAO* | 1.20 [1.10; 1.31] | <0.0001 | 1.10 [1.00; 1.21] | 0.044 | 1.06 [0.96; 1.17] | 0.23 | 1.03 [0.93; 1.14] | 0.54 |
| TMA | 1.05 [0.97; 1.14] | 0.26 | 1.02 [0.94; 1.11] | 0.69 | 1.01 [0.93; 1.10] | 0.81 | 1.01 [0.93; 1.10] | 0.75 |
| Cysteine | 1.34 [1.22; 1.48] | <0.0001 | 1.20 [1.09; 1.32] | 0.0003 | 1.11 [1.00; 1.23] | 0.045 | 1.11 [1.00; 1.24] | 0.041 |
| Homocysteine | 1.30 [1.23; 1.38] | <0.0001 | 1.22 [1.15; 1.30] | <0.0001 | 1.19 [1.10; 1.28] | <0.0001 | 1.18 [1.09; 1.27] | <0.0001 |
| Methionine | 0.96 [0.87; 1.06] | 0.37 | 0.96 [0.87; 1.05] | 0.38 | 0.95 [0.87; 1.05] | 0.32 | 0.99 [0.90; 1.08] | 0.77 |

*TMAO was natural-log transformed before standardization. Cause-specific hazard models were fitted using different adjustment models. All HR are given per 1 SD of the given parameter. M_1_: Model 1, univariate; M_2_: M_1_ covariates + age, sex; M_3A_: M_2_ covariates + history of CAD and log transformed NT-proBPNP; M_3B_: M_2_ covariates + eGFR and log transformed uACR. The nutritional biomarkers were tested separately from each other in the different models.

Abbreviations: CAD: coronary artery disease; CV: cardiovascular; eGFR: estimated glomerular filtration rate calculated with the CKD-EPI 2009-formula; HFrH: Heart Failure requiring Hospitalization, defined as the first occurrence of acute HF leading to hospitalization and/or death; HR: Hazard-ratio; NT-proBNP: N-terminal prohormone of brain natriuretic peptide; TMA: trimethylamine; TMAO: trimethylamine N-oxide; uACR: urine albumin/creatinine ratio
