# Supplementary material for: Nutritional biomarkers and heart failure requiring hospitalization in patients with type 2 diabetes: the SURDIAGENE cohort
Source: Cardiovasc Diabetol. 2022 Jun 9;21:101. doi: 10.1186/s12933-022-01505-9 (PMC9185908; doi:10.1186/s12933-022-01505-9)
Supplement: Supplementary file 3 — Additional file 3. SURDIAGENE ORGANISATION (Committees and staff). [file 12933_2022_1505_MOESM3_ESM.docx]

**SUPPLEMENTARY INFORMATION – SURDIAGENE ORGANISATION
Centers and staff involved in SURDIAGENE recruitment and adjudication
Participants and clinicians**

All participants included and followed in the cohort study are warmly thanked for their kind participation to this research. Their GPs are acknowledged for their help to collect clinical information.

**Center organisation**

Recruiting physicians: Samy Hadjadj (Coordinator), Frédérique Duengler, Louis Labbé, Aurélie Miot, Xavier Piguel, Stéphanie Laugier-Robiole, Florence Torremocha, Pierre-Jean Saulnier, Richard Maréchaud.
Secretarial and technical assistance: Cécile Demer and all the staff from the department of endocrinology, diabetology (recruitment) and Sonia Brishoual and the staff of the INSERM CIC1402 (data management) and Poitiers Biological Resources Center (CRB 0033-00068) (biobanking). We thank Alexandre Pavy, Marie-Claire Pasquier (Information Technology Department, CHU de Poitiers, Poitiers, France) and Ariane Neveu and Julien Guignet (Medical Information Department, CHU de Poitiers, Poitiers, France). Gérard Mauco (Department of biochemistry, CHU Poitiers) and Thierry Hauet (INSERM U1082, CHU Poitiers) are acknowledged for helping in biological determinations.

**Baseline data case review**

All participant records were reviewed to ascertain the following points: type 2 diabetes, diabetic kidney disease, diabetic retinopathy and cardiovascular disease.
The clinicians involved in this process are warmly thanked here: Daniel Herpin & Philippe Sosner (Cardiology), Frank Bridoux (Nephrology), Helene Manic (Ophthalmology) and Samy Hadjadj (Diabetology).

**Adjudication procedure**

Case inquiry – Samy Hadjadj (Coordinator), Sonia Brishoual, Céline Divoy, Cécile Demer, Aurélie Miot, Xavier Piguel, Florence Torremocha, Nathalie Fauvergue, Séverin Carasson, Pierre-Jean Saulnier, Philippe Sosner
Local coordination: Stéphanie Ragot (coordinator & biostatistician), Fabrice Lebel (Data manager), Elise Gand (Data management and biostatistics)

Adjudication committees:

**2009-2013** Jean-Michel Halimi (Chairman, Tours), Gregory Ducrocq (Paris Bichat), Charlotte Hulin (Poitiers), Pierre Llatty (Poitiers), David Montaigne (Lille), Vincent Rigalleau (Bordeaux), Ronan Roussel (Paris Bichat), Philippe Zaoui (Grenoble).

**2015**- Jean Michel Halimi (Chairman Tours), Barnabas Gellen (Poitiers), Philippe Gatault (Tours), Vincent Javaugue (Poitiers), David Montaigne (Lille), Xavier Piguel (Poitiers), Yann Pucheu (Bordeaux), Vincent Rigalleau (Bordeaux), Ronan Roussel (Paris Bichat).

Quality control (INSERM CIC 1402): Pierre-Jean Saulnier, Astrid de Hautecloque, Frederike Limousi, Nathalie Fauvergue, Sofia Hermann, Sonia Brishoual

The members of **SURDIAGENE study group** include Samy Hadjadj, M.D. Ph.D., CHU de Poitiers, l’institut du thorax, Nantes ; Richard Marechaud M.D., CHU de Poitiers ; Stéphanie Ragot, Pharm.D. Ph.D., CHU de Poitiers ; Xavier Piguel, M.D., CHU Poitiers ;Pierre-Jean Saulnier, M.D. Ph.D., CHU de Poitiers ;Vincent Javaugue, M.D. Ph.D., CHU Poitiers ; Elise Gand, M.Sc., CHU Poitiers ; Charlotte Hulin-Delmotte, M.D. ; Pierre Llatty, M.D. ; Gregory Ducrocq, M.D. Ph.D., Assistance Public-Hôpitaux de Paris; Ronan Roussel, M.D. Ph.D., Assistance Publique-Hôpitaux de Paris, M.D. Ph.D. ; Vincent Rigalleau, M.D. Ph.D. , CHU Bordeaux ; Yann Pucheu, M.D., CHU Bordeaux; Philippe Zaoui, M.D. Ph.D. , CHU Grenoble; David Montaigne, M.D. Ph.D. , CHU Lille ; Jean-Michel Halimi, M.D. Ph.D. , CHU Tours ; Philippe Gatault, M.D. Ph.D., CHU Tours; Philippe Sosner, M.D. Ph.D., Laboratoire MOVE EA6314; Barnabas Gellen, M.D. Ph.D., Polyclinique de Poitiers.

**General funding of the cohort**

The SURDIAGENE cohort was supported by grants from the French Ministry of Health (PHRC-Poitiers 2004; PHRC-IR 2008), the Association Française des Diabétiques (Research Grant 2003) and the Groupement pour l’Etude des Maladies Métaboliques et Systémiques (GEMMS Poitiers, France). Additional specific fundings were obtained for dedicated projects.
